# Diagnostic use of ultrasound in giant cell arteritis in Counties Manukau District Health Board, New Zealand

**DOI:** 10.1093/rap/rkac040

**Published:** 2022-05-12

**Authors:** Rathan Nagarajah, Rajiv Gupta, Sunil Kumar

**Affiliations:** Department of Rheumatology, Counties Manukau District Health Board, Middlemore Hospital, Auckland, New Zealand

**Keywords:** GCA, temporal artery biopsy, colour duplex ultrasound, diagnosis

## Abstract

**Objectives:**

A retrospective observational study was undertaken to assess the diagnostic performance (sensitivity and specificity) of colour duplex ultrasound (CDUS) compared with temporal artery biopsy (TAB) for the diagnosis of GCA in the Counties Manukau District Health Board (CMDHB), New Zealand using clinical diagnosis as the reference standard.

**Methods:**

The study population included patients with clinically suspected GCA who were referred to Middlemore Hospital and underwent CDUS, TAB or both between January 2019 and December 2020.

**Results:**

Sixty-nine patients were included in the study. Sixty-one percent were >75 years of age, with no cases <50 years of age and a female predominance of 71%. The sensitivity of CDUS was 26% (95% CI 10, 48) and specificity was 97% (95% CI 84, 100). The sensitivity of TAB was 57% (95% CI 34, 77) and specificity was 100%. CDUS had a positive predictive value of 86% (95% CI 42, 99) and a negative predictive value of 65% (95% CI 49, 78).

**Conclusion:**

A positive CDUS in patients with a high risk for GCA may preclude the need for TAB due to the high specificity of CDUS in GCA. In contrast, patients with a high risk for GCA with a negative CDUS may still need TAB to confirm or exclude GCA. The duration from commencement of steroids to the time of CDUS is crucial in confirming GCA and, for this, shortening the waiting time in the CMDHB would be necessary to ensure adequate test performance in practice.

Key messages

- A positive colour duplex ultrasound (CDUS) in high-risk patients for GCA may preclude the need for temporal artery biopsy (TAB).
- The timing between commencement of steroids and CDUS/TAB is important in establishing a GCA diagnosis.

## Introduction

### Overview of the disease

GCA is a medium and large-vessel vasculitis causing inflammatory and ischaemic changes predominantly in the carotid artery and its extracranial branches [1, 2]. It is most common in people >50 years of age and more commonly reported in Northern Europeans [1–3]. It is a medical emergency and prompt treatment with high-dose steroids is imperative to prevent irreversible, sudden vision loss [3, 4]. The diagnosis is based on the clinical symptoms, signs and laboratory tests as per the 1990 ACR criteria or revised ACR criteria [2]. Temporal artery biopsy (TAB) is the current gold standard for diagnosis of GCA [3, 6]. However, temporal artery colour duplex ultrasound (CDUS) is increasingly being used as a diagnostic test and has shown promising results in recent studies [3, 5–9]. In some centres it is being used as the first-line investigation, replacing TAB, and has been utilized (in conjunction with TAB) in the Counties Manukau District Health Board (CMDHB) since 2019.

### TAB as a diagnostic tool

The characteristic histology from TAB confirms the diagnosis of GCA, but a negative result does not rule it out, owing to false-negative results due to the segmental involvement of the arterial wall [3, 10]. TAB requires an experienced surgeon and may not be easily accessible, resulting in increased wait times. Ideally these investigations should be done within 1 week of initiating treatment with steroids to minimize false-negative results [3, 5].

### CDUS as a diagnostic tool

CDUS is non-invasive, safe and cost-effective, but it is operator dependent. There is evidence that imaging the axillary arteries concurrently with the temporal arteries increases the diagnostic yield of GCA. This is due to persistent changes in larger arteries despite steroid treatment in this multivessel disease [3, 11]. There are four cardinal signs on US to identify arterial wall inflammation: the halo sign (caused by swelling of the middle layer of the arterial wall and seen as a non-compressible hypoechoic band), compression sign, stenosis and occlusion [3, 8, 12, 13]. The halo sign has been shown to have a sensitivity of 69% and 68% and a specificity of 82% and 91% in two metanalyses, respectively [6, 14]. The latter two signs (stenosis and occlusion) are not specific for the disease [7].

## Methods

No written informed consent from the patients was required since this was a retrospective observational study and ethics approval was obtained from the Auckland Health Research Ethics Committee (reference no. AH2870). Locality approval for the study was obtained from the Counties Manukau Health Research Committee (registration no. 1287). The study population included patients with clinically suspected GCA who underwent CDUS, TAB or both between January 2019 and December 2020 at the CMDHB. The patients’ National Health Indexes were identified from the electronic database through Counties Manukau Health informatics using the International Classification of Diseases, 10th revision: diagnosis of GCA or TA (Temporal Arteritis), procedure of temporal artery biopsy or US Doppler other.

Inclusion criteria for the study were patients with clinically suspected GCA who had CDUS of temporal arteries, TAB or both procedures. Exclusion criteria were patients with alternative diagnoses, patients with clinical diagnoses of GCA without the need for CDUS or TAB, those lost to follow-up and patients who were not in the study time frame. Patients who were treated in the private sector (such as through their health insurance providers) were not considered in this study.

Rheumatology clinic letters, inpatient notes, and discharge summaries were reviewed for demographic data (age, gender and ethnicity), presenting symptoms, signs, CRP and ESR. CDUS and histology of TABs were reviewed to confirm the radiological and histological features for the diagnosis of GCA. The date of prednisone commencement, including the dose administered, was recorded to act as a reference point for the time taken to perform CDUS and TAB. This study used clinical diagnosis as the reference standard where clinically positive GCA means a GCA diagnosis given by the treating rheumatologist and clinically negative GCA means unlikely to be GCA as per the rheumatologist. Electronic clinical records of GCA cases were analysed during the first visit (within 1–2 weeks of presentation) and subsequent visits, with the last follow-up in the rheumatology clinic at or just before 6 months from presentation to confirm the clinical diagnosis given by the treating rheumatologist.

### Statistical analysis

All the extracted data were entered into an Excel spreadsheet (Microsoft, Redmond, WA, USA) and a formula was used to calculate the number and proportion of clinically positive and negative GCA cases, CDUS positive and negative cases and biopsy positive and negative cases.

Age, gender and ethnicity-specific incidence rates were calculated for a population of 100 000 >50 years of age in the CMDHB area, with 95% CIs, using the number of incident cases as the numerator and population estimates >50 years of age (based on New Zealand 2013 census data) as the denominator. Chi-squared test or Fisher’s exact test was used to assess differences in age, gender, ethnicity, inflammatory markers, symptoms and signs between the clinically positive and negative GCA cases.

Sensitivities and specificities, along with their 95% CIs, were calculated for CDUS and TAB using the standard reference. The kappa statistic was used to assess the agreement of CDUS and TAB against the clinical diagnosis. The waiting time was reported in terms of median with interquartile range for those with CDUS *vs* TAB for the clinically positive GCA cases. The statistical analysis was performed using SAS version 9.4 (SAS Institute, Cary, NC, USA).

Meta-analysis was also conducted based on 12 international studies to produce pooled estimates of sensitivity and specificity with their corresponding 95% CIs. They were calculated using the DerSimonian and Laird random-effects model. Between-study heterogeneity was assessed using the chi-squared and *I*^2^ tests.

## Results

Seventy-four patients were identified and four were excluded from the study. Two patients were given alternative diagnoses before investigations, one was lost to follow-up and one was not in the study period. One patient had a clinical diagnosis of GCA and was excluded from the study cohort because neither CDUS nor TAB were performed in this case; however, the patient was included in the calculation of data in Tables 1 and 2. The patient was not included in the main analysis for test performance. The remaining 69 patients who underwent either CDUS, TAB or both as a part of their workup for GCA were included in the study cohort.

**Table 1:** Patient demographics, symptoms, signs and inflammatory markers (ESR and CRP) in clinically suspected GCA cases

Characteristics	Clinically positive GCA cases (*n* = 31)	Clinically negative GCA cases (*n* = 39)	Total	*P*-value
Age (years), *n* (%)				
<50	0 (0)	1 (2.6)	1 (1.4)	0.22*
50–64	1 (3.2)	5 (12.8)	6 (8.6)	
65–74	11 (35.5)	17 (43.6)	28 (40)	
>75	19 (61.3)	16 (41)	35 (50)	
Gender, *n* (%)				
Male	9 (29)	12 (30.8)	21 (30)	0.88
Female	22 (71)	27 (69.2)	49 (70)	
Ethnicity, *n* (%)				
New Zealand Europeans/other Europeans	21 (67.7)	24 (61.5)	45 (64.3)	0.89*
Maori	3 (9.7)	3 (7.7)	6 (8.6)	
Pacific Islander	2 (6.5)	4 (10.3)	6 (8.6)	
Chinese	1 (3.2)	3 (7.7)	4 (5.7)	
Indian	2 (6.5)	4 (10.3)	6 (8.6)	
Not stated	2 (6.5)	1 (2.6)	3 (4.3)	
Symptoms, *n* (%)				
Headache	30 (96.8)	33 (84.6)	63 (90)	0.12*
PMR symptoms	18 (58.1)	6 (15.4)	24 (34.3)	0.0002**
Jaw claudication	11 (35.5)	4 (10.3)	15 (21.4)	0.011**
Scalp tenderness	21 (67.7)	14 (35.9)	35 (50)	0.0081**
Non-specific symptoms^a^	8 (25.8)	9 (23.1)	17 (24.3)	0.79
Visual symptoms	16 (51.6)	19 (48.7)	35 (50)	0.81
Visual loss	2 (6.5)	2 (5.1)	4 (5.7)	>0.95*
Signs, *n (%)*				
Fever	1 (3.2)	2 (5.1)	3 (4.3)	>0.95*
Tender temporal artery	14 (45.2)	15 (38.5)	29 (41.4)	0.57
Thickened temporal artery	2 (6.5)	1 (2.6)	3 (4.3)	0.58*
Reduced or absent temporal artery pulse	1 (3.2)	1 (2.6)	2 (2.9)	>0.95*
ESR (mm/h), *n* (%)				
<40	10 (32.3)	20 (51.3)	30 (42.9)	0.024**
40–60	5 (16.1)	11 (28.2)	16 (22.9)	
>60	16 (51.6)	8 (20.5)	24 (34.3)	
CRP (mg/l), *n* (%)				
<5	1 (3.2)	19 (48.7)	20 (28.6)	<0.0001**
5–40	11 (35.5)	12 (30.8)	23 (32.9)	
>40	19 (61.3)	8 (20.5)	27 (38.6)	

a Non-specific symptoms: malaise, anorexia and weight loss.

* Fisher’s exact test used, otherwise the chi-squared test was used.

** *P* < 0.05 as statistically significant.

**Table 2:** Age, gender and ethnicity-specific incidence rates in clinically positive GCA cases

Demographics	*n* (%)	Incidence^a^	95% CI
Age (years)			
50–64	1 (3.2)	0.6	0.02, 3.3
65–74	11 (35.5)	17	8.5, 30.5
>75	19 (61.3)	47	28.3, 73.4
Gender			
Male	9 (29)	6.9	3.2, 13.1
Female	22 (71)	15.5	9.7, 23.5
Total	31 (100)	–	–
Ethnicity			
New Zealand Europeans/other Europeans	21 (67.7)	13.2	8.2, 20.1
Maori	3 (9.7)	12.2	2.5, 35.7
Pacific Islander	2 (6.5)	5.2	0.63, 18.7
Chinese	1 (3.2)	4.3	0.11, 24.1
Indian	2 (6.5)	11.2	1.36, 40.4
Not stated	2 (6.5)	NA	
Total	31	11.4	7.8, 16.7

NA: not available.

a Incidence per 100 000 per year >50 years of age.

Sixty-one percent were >75 years of age, with no cases <50 years of age and a female predominance of 71% (Table 1). The mean annual incidence for this cohort was 11.4 per 100 000 population >50 years of age in the CMDHB area (Table 2). Headache was the most common symptom in GCA positive and negative cases. More than half (58.1%) of the clinically positive GCA cases had a current or past history of polymyalgia rheumatica. Symmetrical polymyalgia, jaw claudication and scalp tenderness were significantly higher in clinically positive than negative GCA cases (statistically significant with a *P*-value <0.05). Elevated ESR (>60 mm/h) and CRP (>40 mg/l) were present in 52% and 61% percent of the clinically positive GCA patients, respectively. Of note, ESR was <40 mm/h in 32% and 3% had a normal CRP in clinically positive cases (Table 1).

**Fig. 1:**
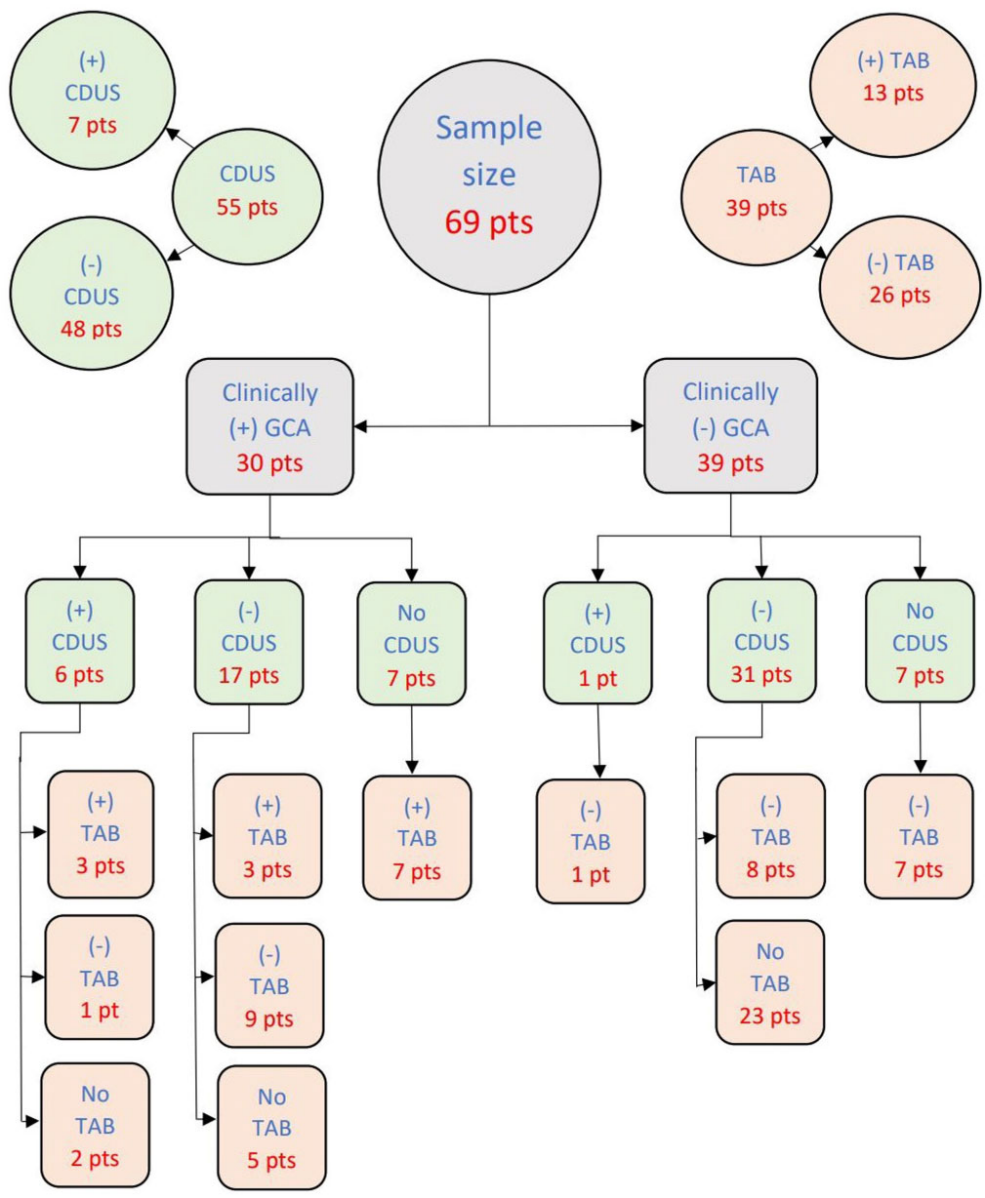
Overview of clinically positive/negative GCA, positive/negative CDUS and positive/negative TAB Pt(s): patient(s).

Of the 69 patients with clinically suspected GCA, 30 patients underwent CDUS, 14 patients had TAB and 25 patients had both CDUS and TAB (i.e. 55 CDUSs and 39 TABs were performed in total). Of the 30 clinically positive GCA cases, 3 patients had both a positive CDUS and TAB, 3 patients had a positive CDUS and 10 patients had a positive TAB. The remaining 14 of 30 clinically positive patients had negative investigations (CDUS/TAB), but GCA was diagnosed clinically based on history, examination and laboratory findings (Fig. 1).

Of the 55 CDUSs in clinically suspected GCA, there were 23 cases with clinically confirmed diagnoses of GCA by the treating rheumatologist (referred to as clinically positive GCA) and 32 cases were not clinically confirmed GCA (referred to as clinically negative GCA). There were 6 positive CDUSs for GCA out of 23 clinically positive GCA cases, resulting in a sensitivity of 26% (95% CI of 10, 48). There were 31 negative CDUSs out of 32 clinically negative GCA cases (1 false-positive CDUS), giving a specificity of 97% (95% CI 84, 100). The positive predictive value and negative predictive value of CDUS for GCA was 86% (95% CI 42, 99) and 65% (95% CI 49, 78), respectively.

Of the 39 patients who underwent TAB, there were 23 cases with clinically positive GCA and 16 cases had a very low clinical probability of GCA (clinically negative GCA). There were 13 histologically proven GCA of the 23 clinically positive GCA patients, giving a sensitivity of 57% (95% CI 34, 77) for TAB in the diagnosis of GCA. The specificity was calculated to be 100%. The Cohen’s kappa for CDUS *vs* a clinical diagnosis of GCA was found to be 0.25 (95% CI 0.05, 0.46), indicating fair agreement, whereas the Cohen’s kappa for TAB *vs* a clinical diagnosis of GCA was 0.52 (95% CI 0.29, 0.74), indicating moderate agreement.

**Fig. 2:**
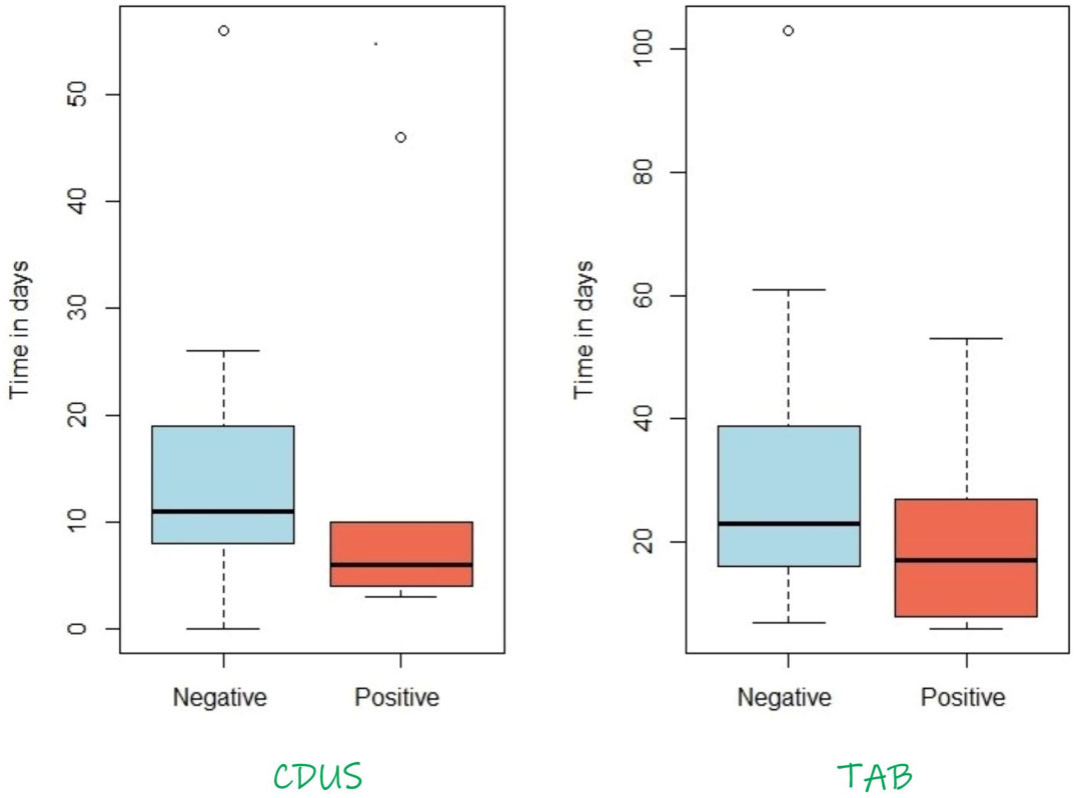
Days from prednisone commencement to CDUS/TAB for patients with clinically positive GCA

For the clinically positive GCA cases, the median time in days from prednisone commencement to CDUS was 6 days [interquartile range (IQR) 4–9.5] in the positive CDUS cases, compared with 11 days (IQR 8–19) in the negative CDUS cases. Similarly, for the clinically positive GCA cases, the median time from prednisone commencement to TAB was much shorter in the positive TAB cases [17 days (IQR 8–27)] compared with the negative TAB cases [23 days (IQR 16.3–38)]. Overall, the negative CDUSs were done later than the ones that were positive for clinically positive GCA patients. There is a similar pattern with TAB reports (Fig. 2).

## Discussion

Demographic data match international data, with the majority of patients being of European origin (68%) and female and all patients being >50 years of age. These findings were similar to an unpublished Waikato study (Quincey V. GCA in the modern era and at Waikato, New Zealand. Internal Medicine Society of Australia and New Zealand (IMSANZ) 2021) and an Otago study in New Zealand [26]. There is a lack of information in the literature regarding the prevalence or incidence of GCA in the Maori population in New Zealand. This observational study analysed the differences in ethnicity data in relation to GCA and found that 9.7% of positive GCA cases were Maori and 6.5% were Pacific Islanders in the CMDHB catchment area. This demonstrates that GCA can manifest in other ethnicities as well.

A 9 year study in Otago, from 1996 to 2005, showed a mean annual incidence of 12.73 per 100 000 for the population >50 years of age. Unpublished data from Waikato (Quincey V. GCA in the modern era and at Waikato, New Zealand. Internal Medicine Society of Australia and New Zealand (IMSANZ) 2021) showed an incidence of 13.6 per 100 000 for the population >50 years of age, which is comparable to this study (11.4 per 100 000 for the population >50 years of age) [26].

**Fig. 3:**
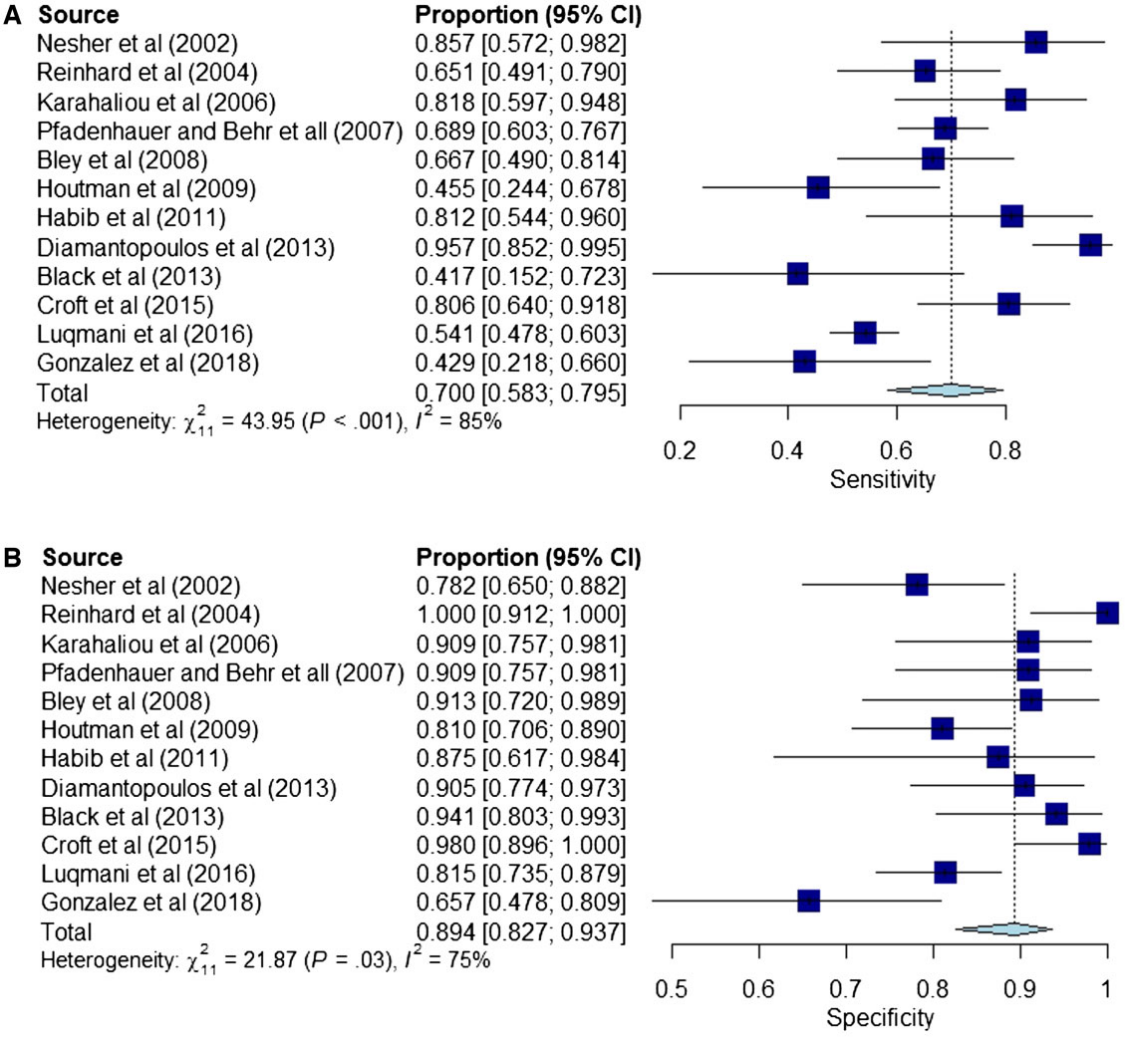
Pooled sensitivity and specificity of CDUS for diagnosing GCA derived from 12 international studies Metanalyses give a pooled sensitivity of 70% (95% CI 58, 79) and a pooled specificity of 89% (95% CI 83, 94).

A review of the literature since 2000 identified papers from five metanalyses that used clinical diagnosis as the reference standard, similar to this study [6–9, 14]. The overall results of these papers showed that CDUS had a higher specificity than sensitivity in the diagnosis of GCA. Sensitivity varied between 42% and 96%, with a pooled sensitivity of 70% (95% CI 58, 79), and the specificity ranged from 65.7% to 100%, with a pooled specificity of 89% (95% CI 83, 94) [3, 10, 15–24] (Fig. 3). There was significant variability between study heterogeneity for sensitivity and specificity. The sensitivity (26%) of CDUS in this study was lower than in the international literature, however, the specificity of CDUS was comparable to the international literature. Local unpublished data from Waikato (Quincey V. GCA in the modern era and at Waikato, New Zealand. Internal Medicine Society of Australia and New Zealand (IMSANZ) 2021) from 2014 to 2019 showed a sensitivity of 32.9% for CDUS in the diagnosis of GCA, which is comparable to our result .

The specificity of 97% in this study was due to one false-positive CDUS. This patient was commenced on high-dose steroids by the general practitioner on suspicion of GCA. Clinical diagnosis of negative GCA was given by the rheumatologist in the first clinic visit in the context of the clinical presentation and lab investigations, despite the positive CDUS. Therefore the patient was rapidly weaned off the steroids. It is important to be mindful of the possibility of a false-positive CDUS and that the diagnosis involves consideration of other factors, including clinical presentation and lab investigations.

There is evidence to show that the sensitivity of CDUS diminishes with the anti-inflammatory effects of corticosteroids. One study showed that there was a significant reduction in the sensitivity of CDUS in diagnosing GCA from the time of commencement of steroids [25]. The results of this study showed that the sensitivity of CDUS decreased from 87.5% on day 1 of steroids to 50% when the patient was on steroids for >4 days. Our study found a median wait time of 6 days for clinically positive GCA cases with positive CDUS. The CDUS results that came back negative for positive GCA cases were done later, with a median of 11 days. This highlights the importance of performing the CDUS soon after commencement of steroids.

As reported in the literature, concurrent CDUS of axillary arteries and temporal arteries improved the sensitivity of CDUS (sensitivity of 52% for CDUS of temporal arteries and 71% for both temporal and axillary CDUS) [11]. Also, negative temporal artery CDUS does not rule out the involvement of the axillary artery or extracranial involvement in GCA [11]. Unfortunately, the majority of patients in this study did not have a CDUS of the axillary artery due to resource constraints.

Only 55 of 69 patients underwent CDUS in this cohort. During the initial phase of induction of CDUS in the CMDHB, all the suspected patients with GCA did not get CDUS. All staff were not aware of the use of CDUS in diagnosing GCA and were following the existing protocol that did not include CDUS for all suspected GCA cases. Six of the 14 patients did not have CDUS for this reason and 8 of the 14 patients did not have CDUS because the management decision by the rheumatologist was based on clinical evaluation and TAB results.

Overall, the study highlights a link between waiting time for investigations (CDUS/TAB) after steroid commencement and its effect on CDUS/TAB findings. Second, this study revealed GCA in the Maori and Pacific Islander populations, suggesting that GCA can occur in ethnicities other than Europeans, with Maori being the second most common population with GCA in the CMDHB catchment area in New Zealand. Finally, it shows there is an urgent need to create a GCA pathway in the CMDHB with the aim of having early CDUS for suspected GCA. Early diagnosis and exclusion of GCA would prevent unnecessary surgical intervention and long-term high-dose steroid treatment and its potential complications.

Limitations of this study were, being a retrospective study, data collection was contingent on good clinical documentation. Also, calculation of the incidence did not include the small proportion of patients treated in the private sector, and this could slightly underestimate the incidence in our study. The wide CIs of our sensitivity and specificity of CDUS and TAB can be attributed to our small sample size. Temporal artery CDUS was recently started as a diagnostic tool for GCA in the CMDHB and operator experience and variability may have influenced the outcome of the test. It is anticipated that the diagnostic performance of CDUS will improve over time. Finally, coronavirus disease 2019 and lockdowns with changes in alert levels in New Zealand during the study period may have influenced access to the investigations for GCA and altered the outcome of this study in terms of waiting times, but this was not investigated in this study.

Moreover, the results of the investigations (CDUS/TAB) may have influenced the treating rheumatologists’ decisions about positive or negative GCA and therefore there may be bias in the clinical diagnosis used as a reference standard in this study. To help overcome this bias, data was reanalysed to see how many clinically positive GCA cases fulfilled standardized diagnosis criteria such as the revised ACR criteria. A total of 27 of 30 clinically positive GCA (90%) cases fulfilled the revised ACR criteria without pathology from a TAB. When pathology was included, 93% of clinically positive GCA cases fulfilled the revised ACR criteria. Lastly, CDUS was not performed consistently on all patients in the sample, thus it could be presumed that patients who did not have CDUS were least suitable for the use of CDUS. This leaves the analysis of test performance open to selection bias. This could be addressed by using a larger prospective study where all patients in the study must have CDUS as part of their investigations. All CDUS and TAB referrals are equally triaged as urgent requests regardless of their probability of having GCA. However, given that the patients did not all receive both CDUS and TAB, there may have been bias in how different patients were selected for each intervention and therefore a risk of confounding by indication.

## Conclusions

CDUS is useful in diagnosing GCA, as it is faster and non-invasive compared with TAB. A positive CDUS in patients with a high risk for GCA may preclude the need for TAB due to the high specificity of CDUS in GCA. Larger prospective studies will be required to further assess the role of CDUS in the diagnosis of GCA. It would be interesting to see if performing CDUS of the axillary artery would yield better results in the CMDHB, due to the high occurrence of extracranial disease in GCA. The time from commencement of steroids to the time of investigation is crucial in confirming GCA, therefore shortening waiting times in the CMDHB is necessary to increase the diagnostic performance of CDUS.

## References

[1] Nesher G , Breuer G. Giant cell arteritis and polymyalgia rheumatica: 2016 update. Rambam Maimonides Med J 2016;7:e0035. 10.5041/RMMJ.10262 PMC5101009 27824543

[2] Sait M , Lepore M, Kwasnicki R et al The 2016 revised ACR criteria for diagnosis of giant cell arteritis – our case series: can this avoid unnecessary temporal artery biopsies? Int J Surg Open 2017;9:19–23.

[3] Luqmani R , Lee E, Singh S et al The role of ultrasound compared to biopsy of temporal arteries in the diagnosis and treatment of giant cell arteritis (TABUL): a diagnostic accuracy and cost-effectiveness study. Health Technol Assess 2016;20:1–238. 10.3310/hta20900 PMC5165283 27925577

[4] Ching J , Smith S, Dasgupta B, Damato E. The role of vascular ultrasound in managing giant cell arteritis in ophthalmology. Surv Ophthalmol 2020;65:218–26. 31775013 10.1016/j.survophthal.2019.11.004

[5] Dejaco C , Ramiro S, Duftner S et al EULAR recommendations for the use of imaging in large vessel vasculitis in clinical practice. Ann Rheum Dis 2018;77:636–43. 29358285 10.1136/annrheumdis-2017-212649

[6] Karassa F , Matsagas M, Schmidt W, Ioannidis J. Meta-analysis: test performance of ultrasonography for giant-cell arteritis. Ann Intern Med 2005;142:359–69. 15738455 10.7326/0003-4819-142-5-200503010-00011

[7] Ball E , Walsh S, Tang T, Gohil R, Clarke J. Role of ultrasonography in the diagnosis of temporal arteritis. Br J Surg 2010;97:1765–71. 20799290 10.1002/bjs.7252

[8] Duftner C , Dejaco C, Sepriano A et al Imaging in diagnosis, outcome prediction and monitoring of large vessel vasculitis: a systematic literature review and meta-analysis informing the EULAR recommendations. RMD Open 2018;4:e000612. 29531788 10.1136/rmdopen-2017-000612 PMC5845406

[9] Rinagel M , Chatelus E, Jousse-Joulin S et al Diagnostic performance of temporal artery ultrasound for the diagnosis of giant cell arteritis: a systematic review and meta-analysis of the literature. Autoimmun Rev 2019;18:56–61. 30408588 10.1016/j.autrev.2018.07.012

[10] Black R , Roach D, Rischmueller M, Lester S, Hill C. The use of temporal artery ultrasound in the diagnosis of giant cell arteritis in routine practice. Int J Rheum Dis 2013;16:352–7. 23981759 10.1111/1756-185X.12108

[11] Hop H , Mulder D, Sandovici M et al Diagnostic value of axillary artery ultrasound in patients with suspected giant cell arteritis. Rheumatology 2020;59:3676–84. 32240306 10.1093/rheumatology/keaa102 PMC7733725

[12] Schmidt W. Ultrasound in the diagnosis and management of giant cell arteritis. Rheumatology (Oxford) 2018;57(Suppl 2):ii22–ii31. 29982780 10.1093/rheumatology/kex461

[13] Monti S , Floris A, Ponte C et al The proposed role of ultrasound in the management of giant cell arteritis in routine clinical practice. Rheumatology (Oxford) 2018;57:112–9. 29045738 10.1093/rheumatology/kex341

[14] Arida A , Kyprianou M, Kanakis M, Sfikakis P. The diagnostic value of ultrasonography-derived edema of the temporal artery wall in giant cell arteritis: a second meta-analysis. BMC Musculoskelet Disord 2010;11:44. 20210989 10.1186/1471-2474-11-44 PMC2837862

[15] Nesher G , Shemesh D, Mates M, Sonnenblick M, Abramowitz HB. Predictive value of the halo sign in color Doppler ultrasonography of the temporal arteries for diagnosing giant cell arteritis. J Rheumatol 2002;29:1224–6. 12064840

[16] Reinhard M , Schmidt D, Hetzel A. Color-coded sonography in suspected temporal arteritis – experiences after 83 cases. Rheumatol Int 2004;24:340–6. 14600785 10.1007/s00296-003-0372-6

[17] Karahaliou M , Vaiopoulos G, Papaspyrou S et al Colour duplex sonography of temporal arteries before decision for biopsy: a prospective study in 55 patients with suspected giant cell arteritis. Arthritis Res Ther 2006;8:R116. 16859533 10.1186/ar2003 PMC1779378

[18] Pfadenhauer K , Behr C. The contribution of ultrasound of the craniocervical arteries to the diagnosis of giant cell arteritis. Clin Ophthalmol 2007;1:461–70. 19668523 PMC2704542

[19] Bley T , Reinhard M, Hauenstein C et al Comparison of duplex sonography and high-resolution magnetic resonance imaging in the diagnosis of giant cell (temporal) arteritis. Arthritis Rheum 2008;58:2574–8. 18668559 10.1002/art.23699

[20] Houtman P , Doorenbos B, Dol J, Bruyn G. Doppler ultrasonography to diagnose temporal arteritis in the setting of a large community hospital. Scand J Rheumatol 2008;37:316–8. 18612936 10.1080/03009740801998804

[21] Habib H , Essa A, Hassan A. Color duplex ultrasonography of temporal arteries: role in diagnosis and follow-up of suspected cases of temporal arteritis. Clin Rheumatol 2012;31:231–7. 21743987 10.1007/s10067-011-1808-0

[22] Diamantopoulos A , Haugeberg G, Hetland H et al Diagnostic value of color Doppler ultrasonography of temporal arteries and large vessels in giant cell arteritis: a consecutive case series. Arthritis Care Res 2014;66:113–9. 10.1002/acr.22178 24106211

[23] Croft A , Thompson N, Duddy M et al Cranial ultrasound for the diagnosis of giant cell arteritis. A retrospective cohort study. J R Coll Physicians Edinb 2015;45:268–72. 27070887 10.4997/JRCPE.2015.403

[24] González Porto SA , Silva Díaz MT, Reguera Arias A et al A comparative study of Doppler ultrasound against temporal artery biopsy in the diagnosis of giant cell arteritis. Reumatol Clín (Engl Ed) 2020;16(5 Pt 1):313–8. 30318270 10.1016/j.reuma.2018.08.007

[25] Hauenstein C , Reinhard M, Geiger J et al Effects of early corticosteroid treatment on magnetic resonance imaging and ultrasonography findings in giant cell arteritis. Rheumatology (Oxford) 2012;51:1999–2003. 22772317 10.1093/rheumatology/kes153

[26] Abdul-Rahman AM , Molteno ACB, Bevin TH. The epidemiology of giant cell arteritis in Otago, New Zealand: a 9-year analysis. N Z Med J 2011;124:44–52. 21475359

